# Identification of peripheral inflammatory markers between normal control and Alzheimer's disease

**DOI:** 10.1186/1471-2377-11-51

**Published:** 2011-05-12

**Authors:** Sam-Moon Kim, Juhee Song, Seungwoo Kim, Changsu Han, Moon Ho Park, Youngho Koh, Sangmee Ahn Jo, Young-Youl Kim

**Affiliations:** 1 Center for Biomedical Science, Division of Brain Diseases, National Institute of Health in Korea (KNIH), Osong Health Technology Administration Complex 643 Yeonje-ri, Gangoe-myeon, Chungcheongbuk-do, 363-951, Republic of Korea; 2 Department of Psychiatry, Korea University Medical College, 516, Gojan-dong, Danwon-gu, Ansan-shi, Gyeonggi-do 425-707, Republic of Korea; 3 Department of Neurology, Korea University Medical College, 516, Gojan-dong, Danwon-gu, Ansan-shi, Gyeonggi-do 425-707, Republic of Korea

**Keywords:** IL-8, biomarker, Alzheimer's

## Abstract

**Background:**

Multiple pathogenic factors may contribute to the pathophysiology of Alzheimer's disease (AD). Peripheral blood markers have been used to assess biochemical changes associated with AD and mild cognitive impairment (MCI) and involved in their pathophysiology.

**Methods:**

Plasma samples and clinical data were obtained from participants in the Ansan Geriatric Study (AGE study). Plasma concentrations of four candidate biomarkers were measured in the normal control (NC), MCI, and AD group: interleukin-8 (IL-8), IL-10, monocyte chemoattractant protein-1 (MCP-1), and tumor necrosis factor-α (TNF-α).

Body mass index (BMI), MMSE (Mini Mental State Examination), CDR(Clinical Dementia Rating) score and homocystein level were recorded with social and demographic information.

**Results:**

Total of 59 subjects were randomly selected for this analysis [NC (n = 21), MCI(n = 20) and AD(n = 18)]. In demographic data, educational year was correlated with the diagnosis states (***p *** < 0.0001). No significant differences in cardiovascular disease, BMI and use of NSAIDs were found in MCI or AD group compared with NC group, respectively. The involvement of inflammatory illness or conditions in subjects, WBC count, fibrinogen and homocystein of the three groups, but no significant differences were found in each groups. The plasma IL-8 level was lower in MCI and AD patients compared with the normal control group (respectively, *p *< 0.0001). The MCI and AD patients had similar MCP-1, IL-10, and TNF-α level.

**Conclusions:**

Our study suggests the existence of an independent and negative relationship between plasma IL-8 levels and functional status in MCI and AD patients.

## 1. Background

Over the past decade, it has become clear that the brain maintains intricate relationships with the immune system. For example, proteins secreted from the brain can regulate physiological processes throughout the body [1]. In Alzheimer's disease (AD), the characteristic amyloid plaques and tangles in the brain are accompanied by prominent local stimulation of innate immune and inflammatory responses [2]. The possibility that inflammatory cytokines play a role in inflammation in the AD brain was initially suggested by the observation that the concentrations of these molecules are increased in AD tissue and are prominently associated with AD lesions [3,4]. The inflammatory cytokines are products of activated microglia and astrocytes, and they stimulate the phagocytotic activity of microglia. The localization of cytokines to activated glial cells has been demonstrated in AD brain tissue by immunohistochemistry [5,6].

Nevertheless, clinical studies of the potential role of inflammation in AD have yielded inconsistent results. Whereas several community-based studies have linked anti-inflammatory interventions to a lowered risk of developing AD [7], a randomized, placebo-controlled clinical trial failed to demonstrate a beneficial effect of nonsteroidal anti-inflammatory drugs (NSAIDs) on the progression of AD [8].

Other observational studies that have evaluated the relationships between markers of systemic inflammation and AD risk have been inconclusive: circulating cytokines have been reported to be elevated [9], decreased [10], or unaltered [11] in AD patients compared with cognitively intact controls. The observed differences might partly be explained by differences in the study populations, such as inclusion criteria or the number of subjects. Nevertheless, the identification of an inflammatory biomarker of AD is required to improve the accuracy of diagnosis and monitor disease progression. In addition, it might be useful diagnostically as an early AD biomarker in combination with other biological markers [12].

Recent evidence suggests that the pathological process in AD begins many decades before the appearance of overt symptoms [13] and that AD rates are predicted to rise substantially in the coming decades [14]. Consequently, non-invasive, readily accessible peripheral biomarkers with a high degree of sensitivity and specificity would be ideal for screening at-risk individuals. In this study, we examined whether cytokines could be potent biomarkers for diagnosing AD. We measured the levels of four candidate biomarkers (IL-8, IL-10, MCP-1 and TNF-α) in plasma samples and compared them with the risk of developing AD in Asian subjects.

## 2. Methods

### Study population

Plasma cytokine levels and clinical data were obtained from participants in the Ansan Geriatric Study (AGE study) [15,16]. A total of 1,391 subjects (595 men and 796 women) were randomly recruited between September 2004 and March 2006. The follow-up assessment occurred from 2006 to 2008 (second-wave study; 25.61 ± 5.08 months), with uniform, structured follow-up evaluations performed by examiners who were blinded to the collected data. In all, 841 subjects were recruited randomly from the first-wave study. The second follow-up sample (n = 600) was recruited between 2008 and 2009. Among the second follow-up group, total of 59 subjects were randomly selected for this analysis. Informed written consent for participation was obtained from each individual, and the study protocol was approved by the institutional review board of the AGE study. All subjects with recent infections or myocardial infarction or who had undergone antiplatelet, antihypertensive, antineoplastic, or immunosuppressive drug treatments were excluded from the study.

### Diagnosis of dementia

Dementia was defined according to the diagnostic features of dementia given in the Diagnostic and Statistical Manual of Mental Disorders, fourth edition (DSM-IV) [17]. The sub-diagnosis of possible or probable Alzheimer's disease was based on the National Institute of Neurological and Communicative Disease and Stroke-Alzheimer's Disease and Related Disorder Association (NINCDS-ADRDA) criteria [18]. The diagnosis of mild cognitive impairment (MCI) was made in accordance with the clinical criteria of Peterson *et al*. [19].

### Measurements of plasma cytokine concentration

Blood samples were taken from participants after an overnight fast. Plasma was separated from the blood samples, and the cytokine concentrations were measured. We measured IL-8, IL-10, MCP-1, and TNF-α using the BioPlex cytokine assay (Human Group I assay panel, Bio-Rad, Veenendaal, The Netherlands). In addition, we quantified the plasma IL-8, IL-10, MCP-1, and TNF-α levels using enzyme-linked immunosorbent assays (ELISAs) to confirm the BioPlex data. ELISAs were carried out with the Human ELISA kit (R&D Systems, Minneapolis, MN, USA) according to the manufacturer's instructions.

### COX-2 determination by western blot

Platelet proteins were separated in 10% SDS-PAGE and electroblotted to PVDF membranes in a buffer containing 0.025 M Tris-HCl, 0.192 M glycine, pH 8.3, at 230 mA for 2 h and 30 min. After blocking with 10% non-fat milk, immunostaining reaction was performed with a polyclonal antibody raised against N-terminal of COX-2 (N-20; Santa Cruz Biotechnology). A peroxidase conjugate secondary antibody was used (Santa Cruz Biotechnology). The concentration of the protein was determined by Bio-Rad Protein Assay (Bio-Rad). Equivalent amounts of protein (100 μg) were fractionated on 10% SDS polyacrylamide gel overnight, and proteins were transferred to nitrocellulose membranes under semidry conditions. After incubation, the band intensities were evaluated by bioimaging system (MultiGenius, Syngene, USA) and bands were quantified on digitized images.

### Statistical analyses

Cytokines levels were reported as the mean ± standard deviation (SD). Independent chi-square test and one-way analysis of variance (ANOVA) were used to compare diagnosis states. The results were reported separately for each group (AD patients and control subjects). Statistical analysis was performed using SAS ver. 9.1 (SAS Institute, Cary, NC, USA).

## 3. Results

**Figure 1:**
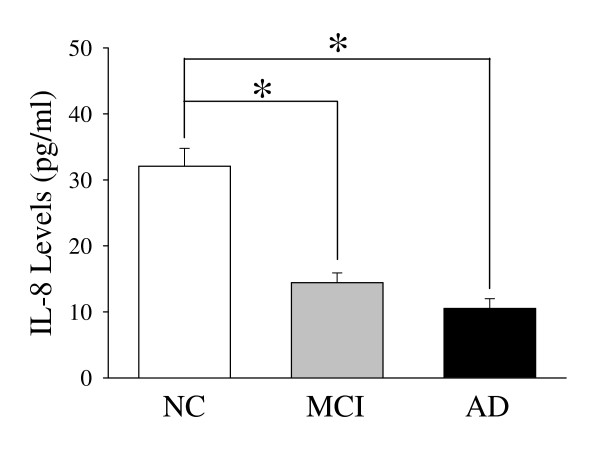
**Plasma levels of IL-8 cytokine from Control (n = 21), MCI (n = 20), and Alzheimer's patients (n = 18)**. Results are expressed as the mean ± SD. Differences statistically significant between diagnosis states (* *p *< 0.0001).

**Figure 2:**
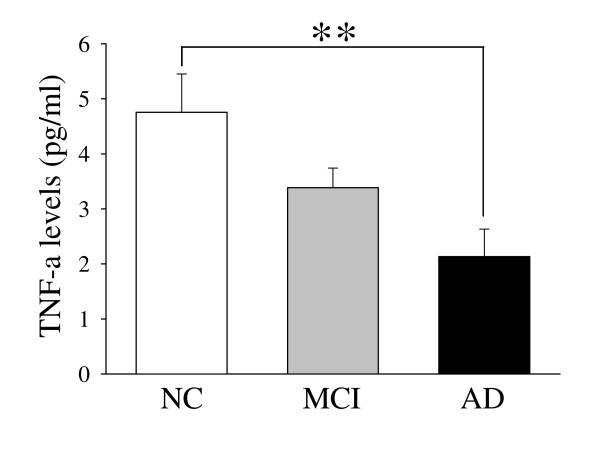
**Plasma levels of TNF-α cytokine from Control, MCI, and Alzheimer's patients**. Results are expressed as the mean ± SD. Differences statistically significant between diagnosis states (** *p *= 0.005).

**Figure 3:**
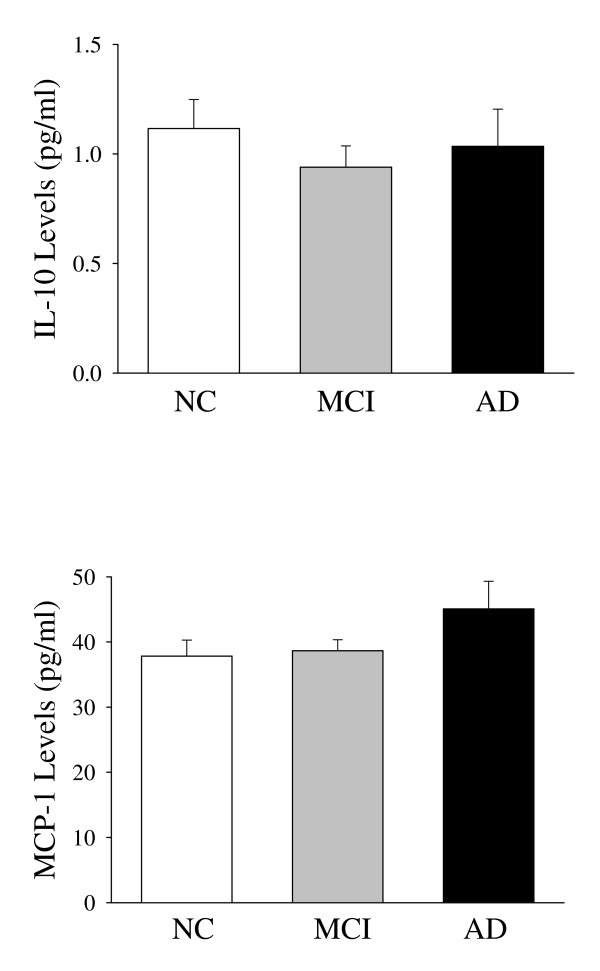
**Plasma levels of IL-10 and MCP-1 from control, MCI, and Alzheimer's patients**. Results are expressed as the mean ± SD.

Table 1 summarizes the demographic factors and clinical characteristics of individual in the normal control (NC), MCI, and AD group. The three groups were similar with respect to age and gender. Educational year was correlated with the diagnosis states (***p *** <0.0001). However, no significant differences in cardiovascular disease, BMI and use of NSAIDs were found in MCI or AD group compared with NC group, respectively. We also compared the involvement of inflammatory illness or conditions in subjects, WBC count, fibrinogen and homocystein of the three groups, but no significant differences were found. IL-8 concentrations are related to MMSE, disease stage or progression, negatively. The plasma IL-8 levels in the three study groups are given in Figure 1. The circulating plasma IL-8 levels were higher in controls than in MCI and AD patients (respectively, *p *< 0.0001). The TNF-α levels were higher in controls compared to AD patients (*p *= 0.005). The plasma TNF-α levels were higher in the control group than in the MCI patients, but the difference was not significant (Figure 2). The plasma IL-10 and MCP-1 levels did not differ among the groups (Figure 3).

**Table 1:** Comparison of the demographic characteristics and dementia-related scale scores of the participants

	NC (n = 21)	MCI (n = 20)	AD (n = 18)	*p*-value	*Tukey HSD
**Age**	75.5 ± 1.3	76.1 ± 2.8	75.9 ± 6.0	0.884	
**Gender**				0.954	
Male (%)	10 (47.6)	9 (45.0)	9 (50.0)		
Female (%)	11 (52.4)	11 (55.0)	9 (50.0)		
**Educational level (y)**	11.6 ± 3.8	6.6 ± 4.9	4.7 ± 4.1	<0.0001	a > b = c
**BMI (%)**	24.7 ±.2.0	24.9 ± 3.0	22.7 ± 2.3	0.016	a = b > c
**CVD (%)**	38.1	70.0	33.3	0.044	
**NSAIDs use (%)**	19.1	5.0	11.1	0.423	
**MMSE**	27.9 ± 1.6	24.3 ± 2.6	15.9 ± 6.1	<0.0001	a > b > c
**CDR score**	0	0.1 ± 0.2	1.1 ± 0.8	<0.0001	a < b = c
**GDS score**	6.4 ± 3.5	7.2 ± 4.3	8.1 ± 6.7	0.547	a = b = c
**WBC (1000/ul)**	5.7 ± 1.3	6.4 ± 1.3	6.9 ± 1.4	0.046	a = b < c
**Fibrinogen (mg/dL)**	318.8 ± 52.4	351.8 ± 67.8	356.3 ± 107.7	0.252	a = b = c
**Homocystein (umol)**	15.9 ± 5.5	17.7 ± 4.8	19.4 ± 10.7	0.339	a = b = c

Data are expressed as means ± standard deviation unless otherwise indicated.

Independent χ^2 ^test and one-way analysis of variance (ANOVA) were used to compare differences between diagnosis states. *Tukey's Studentized Range (HSD) Test

NC: Normal control.

MCI: Mild cognitive impairment.

AD: Alzheimer disease.

BMI: Body mass index

CVD: Cardiovascular diseases

NSAIDs: Non-steroidal anti-inflammatory drugs

MMSE: Mini Mental State Examination

CDR: Clinical Dementia Rating

WBC: White Blood Cell

**Figure 4:**
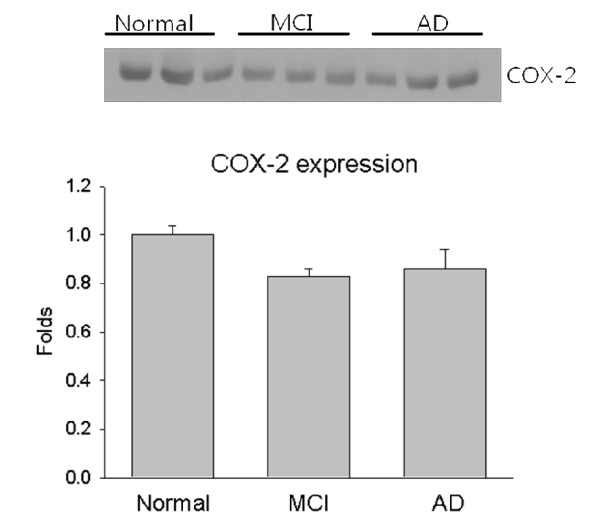
**Representative Western blot of COX-2 in controls (1-3 lanes), in patient with MCI (4-6 lanes) and in Alzheimer group (7-9 lanes)**.

Inflammatory process is induced cyclooxygenase-2 (COX-2). The expression of COX-2 is observed a representative western blot in each group (Figure 4). The expression of COX-2 was not changed in patients with MCI and patients with AD versus control group.

## 4. Discussion

This study evaluated peripheral markers of inflammation in elderly patients with MCI or AD and in normal elderly subjects to assess biochemical changes associated with AD and MCI and involved in their pathophysiology.

Interleukin-8, a chemokine produced by macrophage response to proinflammatory mediators such as amyloid, could be important for recruiting activated microglia into sites of the brain damaged by AD [20]. CXCR2, IL-8 receptor, has been localized to dystrophic neurites, suggesting that IL-8 mediates glial interactions with neurons and thereby contributes to neuronal damage [21]. IL-8 was significantly increased in the cerebrospinal fluid (CSF) in AD compared to controls [22], whereas the plasma IL-8 level in late-onset AD and vascular dementia did not differ from controls in the European subjects [23]. By contrast, our data showed that the IL-8 concentration was significantly lower in patients with MCI or AD compared with the controls. In addition, the ethnic difference of cytokine levels in plasma may exist between Asian subjects and European subjects [24]. However, the levels of COX-2 in the three studied groups have been detected, which could not indicate that it is an enzyme which might be induced with cytokine level. Nevertheless, its diagnostic usefulness is shown a dynamic fluctuation between normal control and AD group. To our knowledge, this is the first report of a negative relationship between IL-8 plasma levels and functional status in older individuals affected by AD.

Tumor necrosis factor alpha is a nonspecific, but potent, factor in the development of several psychiatric diseases, including depression and dementia [25]. In the pathogenesis of AD, TNF-α is produced by activated microglia, mainly in response to the Aβ(1-40) and Aβ(1-42) peptides, as well as to oxidative stress [20]. Although the serum concentrations of TNF-α and the soluble TNF-α receptor increase with age [26,27], results regarding the serum TNF-α concentrations in patients with AD are inconsistent [28-30]. We found decreased TNF-α levels in the plasma from patients with AD compared with the healthy elderly subjects. We observed a similar decrease in this biomarker in the MCI group, but the decrease was not statistically significant compared to the controls.

Monocyte chemoattractant protein-1 is produced by microglial cells and stimulates astrocytes, which together participate in the degradation of Aβ peptides [20]. Significantly increased MCP-1 levels were found in MCI and mild AD, but not in severe AD patients as compared with controls [31], and evidence indicates that the plasma MCP-1 levels could serve as biomarkers to monitor the inflammatory process of AD [32]. We found elevated MCP-1 levels in the plasma from AD patients compared with the healthy elderly subjects, but this increase was not significant.

Interleukin-10 is an anti-inflammatory cytokine in the central nervous system (CNS) that may function to reduce inflammation in AD. However, patients with dementia are reported to have higher mean levels of IL-10 [33], and an increase in brain IL-10 has been reported in neurological disease, including AD [34]. In this study, IL-10 was the same in the plasma of AD patients and controls.

The involvement of cytokines in AD is inferred from several changes in their concentrations in both CSF and plasma [35,36]. Although whether certain biomarkers from the brain enter the circulation or vice versa is still debated, either microglia or other peripheral cells produce and secrete a wide range of cytokines and chemokines [37]. Circulating cytokines have short half-lives, they may reach high concentrations at the sites of release and much lower concentrations after dilution in blood, and they may circulate bound to molecules that can prevent their detection by immunological methods [38]. All of these may contribute to the great variability in the reported data.

The analysis of these cytokines in these subjects, not available in severe AD cases, would not allow us to determine if these alterations are related to the progression or the severity of the disease. Even though these data are not sufficient to show a trend of cytokine level's alteration according to the progression of disease, it would be served as preliminary data to develop inflammatory biomarker for AD diagnosis.

## 5. Conclusions

The circulating plasma IL-8 levels were higher in controls than in MCI and AD patients (respectively, *p *< 0.0001). However, the levels of COX-2 in the three studied groups have been detected, which could not indicate that it is an enzyme which might be induced with cytokine level. Nevertheless, its diagnostic usefulness is shown a dynamic fluctuation between normal control and AD group.

## Competing interests

The authors declare that they have no competing interests.

## Authors' contributions

SM and SW carried out the immunoassay. JH performed the statistical analysis. CS and MH participated in cohort study. YH and SM participated in its design and coordination.

YY conceived of the study, and participated in its design and coordination. All authors read and approved the final manuscript.

## Pre-publication history

The pre-publication history for this paper can be accessed here:

http://www.biomedcentral.com/1471-2377/11/51/prepub
